# MicroRNA-989 targets *5-hydroxytryptamine receptor1* to regulate ovarian development and eggs production in *Culex pipiens pallens*

**DOI:** 10.1186/s13071-023-05957-0

**Published:** 2023-09-13

**Authors:** Junnan Zheng, Jingwei Xu, Ruiming Zhang, Jiajia Du, Huan Wang, Jinze Li, Dan Zhou, Yan Sun, Bo Shen

**Affiliations:** 1 Department of Clinical Laboratory, Huai’an TCM Hospital Affiliated to Nanjing University of Chinese Medicine, Huai’an, 223001 Jiangsu People’s Republic of China; 2 Department of Pathogen Biology, Nanjing Medical University, Nanjing, 211166 Jiangsu People’s Republic of China

**Keywords:** MicroRNA, 5-hydroxytryptamine, Reproduction, Mosquito

## Abstract

**Background:**

Female mosquitoes need a blood meal after mating for their eggs to develop, and this behavior leads to the spread of pathogens. Therefore, understanding the molecular regulation of reproduction in female mosquitoes is essential to control mosquito vector populations. In this study, we reported that microRNA-989 (miR-989), which targets *5-HTR1* (encoding secreted *5-hydroxytryptamine receptor1*), is essential for mosquito reproduction.

**Methods:**

The spatiotemporal expression profile of miR-989 was detected using quantitative real-time reverse transcription PCR (RT-qPCR). miR-989 antagomirs and antagomir-negative control (NC) were designed and synthesized to knock down the expression of endogenous miR-989 in female mosquitoes. RNA sequencing was used to analyze the ovarian response to miR-989 deletion. The targets of miR-989 were predicted and confirmed using RNAhybrid and dual-luciferase assays.

**Results:**

miR-989 is exclusively expressed in female mosquito ovaries and responds to blood feeding. Injection of the miR-989 antagomir resulted in smaller ovaries and reduced egg production. *5-HTR1* was demonstrated as a target of miR-989. The deletion of miR-989 contributed to the upregulation of *5-HTR1* expression. Knockdown of *5-HTR1* rescued the adverse egg production caused by miR-989 silencing. Thus, miR-989 might play an essential role in female reproduction by targeting *5-HTR1*.

**Conclusions:**

We found that miR-989 targets *5-HTR1* and participates in the regulation of reproduction in female mosquitoes. These findings expand our understanding of reproduction-related miRNAs and promote new control strategies for mosquitoes.

**Graphical Abstract:**

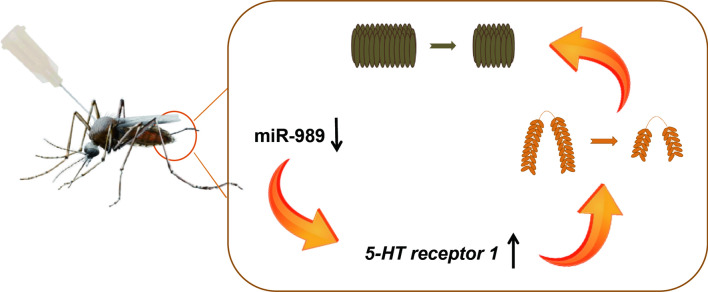

**Supplementary Information:**

The online version contains supplementary material available at 10.1186/s13071-023-05957-0.

## Background

More than 14,000 distinct insect species engage in hematophagy [1]. The repeated blood-feeding behavior of anautogenous female mosquitoes is directly linked to the transmission of mosquito-borne diseases. *Culex pipiens pallens* is the principal vector of West Nile disease, lymphatic filariasis, and Saint Louis encephalitis [2, 3]. The vitellogenesis of mosquitoes, which is activated by a blood meal, includes the synthesis, transmission, and storage of the nutrients essential for embryonic development. These processes are regulated by a complex gene regulation network and have been extensively studied [4–6]. Targeting mosquito vitellogenesis to reduce the vector population could be a promising strategy to prevent mosquito-borne diseases.

MicroRNAs (miRNAs) are a class of non-coding RNA molecules of 21–23 nucleotide length, which bind to the target mRNA and inhibit posttranscriptional gene expression [7, 8]. miRNAs are widely present in insects, and numerous studies have shown their distinctive functions in a variety of crucial developmental events, such as metamorphosis [9–11], reproduction [12, 13], and metabolism [14]. The metabolic balance of miR-276 has been demonstrated to influence the reproduction and *Plasmodium falciparum* growth in *Anopheles coluzzii* [15]. MicroRNA-277 modulates lipolysis and reproduction in *Aedes aegypti* by targeting *ILP7* (encoding insulin-like peptide 7) and *ILP8* [14]. miR-8, miR-275, and miR-1174 are required for blood digestion and oogenesis in *Ae. aegypti* [16–18]. Investigating female mosquito miRNA function is critical to a comprehensive understanding of the genetic regulation of mosquito reproduction and to provide a theoretical basis for making full use of these small molecules.

miR-989 was shown to be specifically highly expressed in female insect [19]. In previous studies, miR-989 was shown to be associated with several key physiological processes in insects. miR-989 mutants produce significant defects in border cell migration in *Drosophila melanogaster* [20]. MiR-989 is significantly expressed in the ovary of *Anopheles gambiae*, which was validated using a microarray [21] and small RNA sequencing investigations [22]. Furthermore, at 24 h post blood meal (PBM), representing the peak of vitellogenesis, the expression of miR-989 was upregulated compared with that before blood feeding, suggesting that miR-989 might play an important role in female mosquito reproduction [23]. Despite being a highly expressed miRNA in the mosquito ovary, the function and target of miR-989 have not been clarified. Therefore, our investigation proposed to explore the function of miR-989 in ovarian development and egg production in female mosquitoes. The abnormal ovarian growth and oviposition responses to miR-989 depletion confirmed that it participates in programming correct ovarian development. We performed RNA sequencing (RNA-seq) to analyze the response of the whole genome to miR-989 depletion and experimentally verified *5-HTR1* (encoding secreted 5-HT receptor 1) as a real target of miR-989. Our results might provide a novel strategy to control the density of mosquito populations.

## Methods

### Mosquito rearing

The laboratory strain of *Cx. pipiens pallens* was collected in 2009 from the Jiangsu Province town of Wuxi and raised in our laboratory without exposure to any insecticides. Mosquito larvae were reared in dechlorinated tap water supplemented with rat chow. Adult mosquitoes were provided with 5% glucose solution and water at 28 ± 1 °C, with a 14:10 h light/dark cycle, and 70–80% relative humidity until blood was sucked. Female mosquitoes were fed with the fresh blood of mice at 3 to 4 days after fledging to stimulate egg production.

### RNA isolation and quantitative real-time reverse transcription PCR (RT-qPCR)

Total RNA was isolated from ovaries or other tissues using the RNAiso Plus reagent (Takara, Dalian, China) following the manufacturer’s protocol. The RNA quality and quantity were assessed using a NanoDrop 2000 instrument (Thermo Fisher Scientific, Waltham, MA, USA). cDNA was produced from 1 μg total RNA using a PrimeScript™ RT Master Mix (Takara) and PrimeScript RT Reagent Kit (Takara). The cDNA was diluted 1:10, and 4 μl of the diluted cDNA solution was used as template for quantitative real-time PCR (RT-qPCR) using the BlasTaq™ 2X qPCR MasterMix (ABM, Vancouver, Canada). Gene-specific qPCR primers were designed using Primer Premier 6.0 software (Premier Biosoft, Palo Alto, CA, USA) (Additional file 1: Table S1). Melting curve analysis was then carried out on a CFX96™Real-Time PCR System (BIO-RAD, Hercules, CA, USA). Expression of miR-989 was determined by the stem-loop method [24]. Specific forward primers and universal reverse primers (URP) were used in the qPCR reactions for miRNAs. The cycle threshold (Ct) values of U6 and *ACTB* (β-actin) were employed to standardize the miR-989 and *5-HTR1* Ct values. Quantitative measurements were carried out using three replicates, and the relative expression (RE) was calculated as RE = 2−ΔCt [25]; *P* values were obtained using Student’s t-test.

### Antagomir and short interfering RNA (siRNA) treatments

Antagomirs were designed and purchased from GenePharma (GenePharma, Shanghai, China). A siRNA was designed and synthesized by GenePharma according to the open reading frame (ORF) of *5-HTR1* (CPIJ015747) (Additional file 1: Table S2). At 24 h post-eclosion (PE), 50 pmol of antagomirs or siRNA was microinjected into female mosquitoes. Mosquitoes were provided 3–4 days to recuperate before being dissected at 24 h PBM.

### RNA-Seq

Ovary samples were collected from wild-type or Ant-989-injected mosquitoes. Ovaries were dissected in sterile 1 × phosphate-buffered saline (PBS), immediately plunged into liquid nitrogen and removed into a -80℃ refrigerator 30 min later until they were shipped to Personal Biotechnology Co. (Shanghai, China) for RNA sequencing. For each group, ovaries were taken from roughly 60 female mosquitoes. Following total RNA extraction and DNase I treatment, Oligo(dT) magnetic beads were used to enrich for mRNA having polyA structure. RNA was split into around 300-bp-long fragments by ion interruption. Using six-base random primers and reverse transcriptase, the first-strand cDNA was created using RNA as the template, and the second-strand cDNA was created using the first-strand cDNA as a template. Following library preparation, PCR amplification was used to enrich the library fragments, and libraries were selected based on fragment size (450 bp). Then, the Agilent 2100 Bioanalyser (Agilent, Santa Clara, CA, USA) was used to assess library quality and detect library concentrations. After RNA extraction, purification, and library construction, libraries were sequenced using next-generation sequencing (NGS) based on the Illumina sequencing platform with paired-end (PE) sequencing. Quality control was performed on the raw reads generated by the Illumina Novaseq6000 platform (Illumina Inc. San Diego, CA, USA) to exclude adapters, unidentified biases, and low-quality reads.

### Bioinformatic analysis and target prediction

The clean reads were aligned with reference genes of *Cx. quinquefasciatus* (https://www.vectorbase.org/organisms/culex-quinquefasciatus) by using BWT algorithms [26]. We used the HISAT2 software for comparisons, and the parameters are shown in the Supplementary Material (Additional file 2). Gene expression levels were calculated using the fragments per kilobase of exon per million mapped fragment (FPKM) method [27], which minimized differences in gene length and sequencing, and was applied for the comparison of differentially expressed genes (DEGs). To determine the potential gene targets of miR-989, we used the RNAhybrid program to screen genes from among the upregulated DEGs that are complementary to the seed region of miR-989 [28].

### Vector construction

Using the specific primers listed in Table 1, the region of the *5-HTR1* 3′ untranslated region (UTR) (*5-HTR1* 3′UTR-wild-type (WT)) and mutant 3′ UTR (*5-HTR1* 3′UTR-Δ) were amplified from the cDNA of *Cx. pipiens pallens*. The primers were created using the genome of the closely related *Cx. quinquefasciatus*. The corresponding sequence of the miR-989 seed region was present in the region of the *5-HTR1* 3′ UTR, and four bases in the seed region were altered in *5-HTR1* 3′ UTR-Δ. The *5-HTR1* 3′ UTR and *5-HTR1* 3′ UTR-Δ sequences were both cloned into the pMIR-REPORT™ miRNA Expression Reporter Vector (Ambion, Austin, TX, USA) using the Hind III and Sac I sites. The pMIR-*5-HTR1*-UTR and pMIR-*5-HTR1*-MUT constructs were verified by sequencing.

**Table 1:** RNA sequencing (RNA-Seq) map statistics

Sample	Clean_Reads	Total_Mapped	Multiple_Mapped	Uniquely_Mapped
WT1	40,891,636	29,133,756 (71.25%)	1,367,735 (4.69%)	27,766,021 (95.31%)
WT 2	42,717,158	30,277,181 (70.88%)	1,467,620 (4.85%)	28,809,561 (95.15%)
WT 3	42,630,186	30,270,115 (71.01%)	1,489,950 (4.92%)	28,780,165 (95.08%)
Ant-989 1	38,437,018	27,436,439 (71.38%)	1,224,010 (4.46%)	26,212,429 (95.54%)
Ant-989 2	38,934,162	27,933,835 (71.75%)	1,242,457 (4.45%)	26,691,378 (95.55%)
Ant-989 3	44,365,454	31,490,768 (70.98%)	1,465,975 (4.66%)	30,024,793 (95.34%)

### Cell culture and dual-luciferase assay

HEK293T cells were cultured in Dulbecco’s Modification of Eagle’s Medium (Bio channel, Nanjing, China) containing 10% fetal bovine serum and 1% penicillin-streptomycin solution at 37 °C. Cells (5 × 10^4^ cells/well) were seeded into 24-well plates and grown to 60–70% confluence (usually 24 h) before transfection. The cells were transfected with recombinant plasmids (100 ng), pGL4.74 plasmids (control reporter, 50 ng), and 20 μm miR-989 mimic or NC per well using Lipofectamine 2000 (Invitrogen, Waltham, MA, USA). The sequences of the miR-989 mimic and NC are shown in Table 2; both were synthesized from GenePharma. Cells were collected and split at 36 h after transfection, and luciferase activities were measured using the Dual-Luciferase Assay (Promega, Madison, WI, USA). Normalized firefly luciferase activity (firefly luciferase activity/Renilla luciferase activity) was compared with that of the control. Each sample was assessed in triplicate.

**Table 2:** Genes upregulated after interfering with miR-989

Gene	Transcript ID	Foldchange (Ant-989/WT)
Cytochrome P450 325BD1	CPIJ015960	8.220622523
Alpha-tropomyosin 5a	CPIJ002405	5.731569036
Vitamin K-dependent protein C precursor	CPIJ018737	5.374109794
Pre-mRNA splicing factor 17 variant	CPIJ015468	4.682345051
Six/sine homebox transcription factors	CPIJ010064	4.371979522
Thrombospondin-4	CPIJ016497	4.332154738
5-Hydroxytryptamine receptor 1	CPIJ015747	4.22338887
Thrombospondin	CPIJ016498	3.558322229
Centrin	CPIJ011558	3.168007909
Integrin alpha-PS5 precursor	CPIJ002982	3.167624358

### Database submission

All high-throughput sequencing data were deposited in the NCBI Sequence Read Archive (SRA) database (accession number: PRJNA972898).

### Statistical analysis

Each statistical value is presented as mean ± SEM of three separate experiments. Means were compared using Student’s t-test at the following significance levels: *P < 0.05, **P < 0.01, and ***P < 0.001.

## Results

### Spatiotemporal expression profile of miR-989 in female mosquitoes

**Fig. 1:**
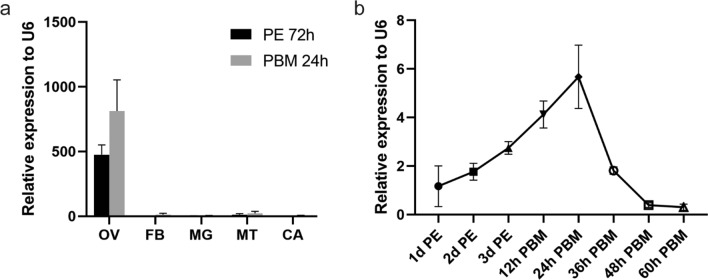
Spatiotemporal expression profile of miR-989. **a** Relative expression of miR-989 was detected in the ovary (OV), fat body (FB), midgut (MG), Malpighian tubule (MT), and carcass (CA) at 72 h post eclosion (PE) and 24 h post blood meal (PBM). **b** Relative expression of miR-989 in female mosquitoes analyzed at 1, 2, and 3 days PE and at 12, 24, 36, 48, and 72 h PBM. Data represent three biological replicates and three technical replicates and are shown as mean ± SD

miR-989 was observed to be highly expressed in female mosquito ovaries. Therefore, we first detected the expression of miR-989 in different tissues of female mosquitoes and found that miR-989 was specifically expressed in the ovary. In other tissues (midgut, fat body, Malpighian tubule, and carcass), its levels were low both before and after a blood meal (Fig. 1a). We subsequently detected the expression of miR-989 in the female mosquitoes over the first reproductive cycle. The abundance of miR-989 was relatively low when the female mosquitoes first fledged and then continued to increase, reaching its peak at 24 h PBM, with 4.86-fold higher expression compared with that at first fledge, before decreasing to 3.13-fold at 36 h PBM. Its expression was almost undetectable at 48 h PBM (Fig. 1b). These results suggested that female mosquito reproduction might be significantly influenced by the high expression of miR-989 in the ovary.

### miR-989 suppression causes abnormal ovarian development and egg production

**Fig. 2:**
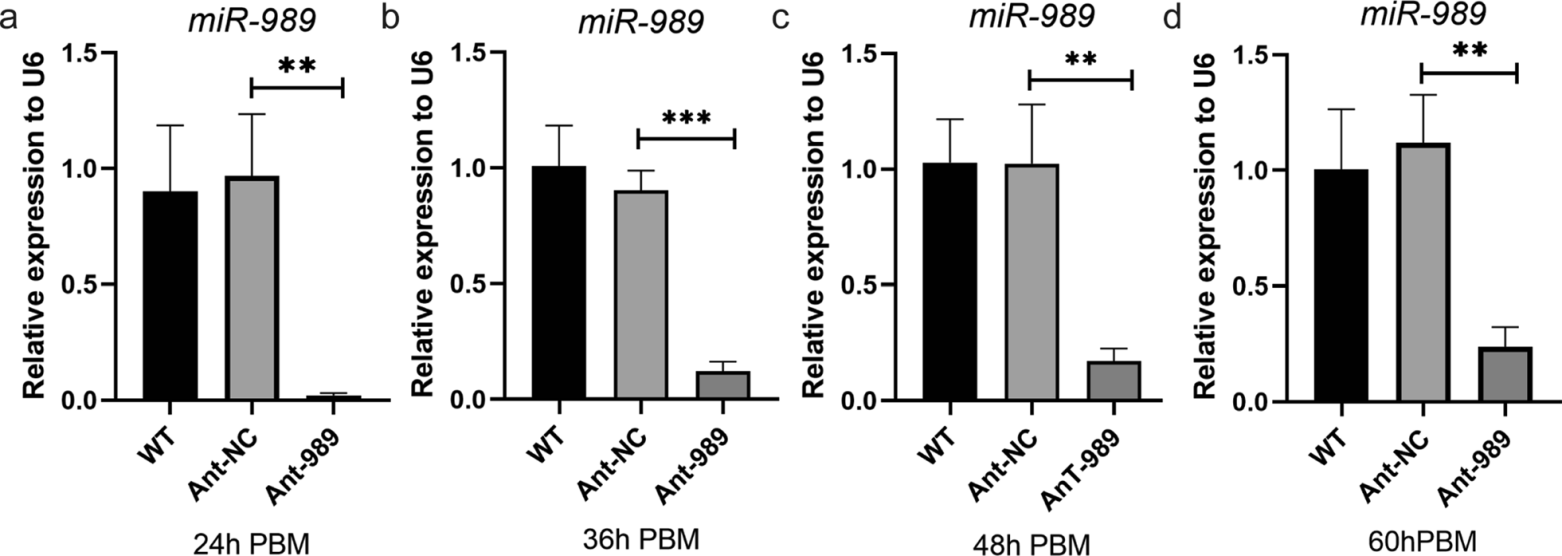
Expression of miR-989 in WT, Ant-NC, and Ant-989 groups of female mosquitoes. **a** Expression of miR-989 in each group at 24 h PBM. **b** Expression of miR-989 in each group at 36 h PBM. **c** Expression of miR-989 in each group at 24 h PBM. **d** Expression of miR-989 in each group at 24 h PBM. Data represent three biological replicates and three technical replicates and are shown as mean ± SD

To further investigate the role of miR-989 in female mosquito reproduction, we designed the miR-989 sequence-specific antagonists (Ant-989), comprising chemically modified antisense oligonucleotides, to knock down endogenous miR-989 in vivo. A scrambled oligonucleotide sequence antagomir (Ant-NC) was also designed as a negative control. Each mosquito was microinjected with Ant-989 or Ant-NC at 12 h PE. Compared with the Ant-NC group, expression of miR-989 in the Ant-989 group was decreased by 96.67% (Student’s t-test: *t*_(4)_ = 6.131, *P* = 0.0036), 86.72% (Student’s t-test: *t*_(4)_ = 14.2, *P* = 0.0001), 83.41% (Student’s t-test: *t*_(4)_ = 5.646, *P* = 0.0048) and 78.87% (Student’s t-test: *t*_(4)_ = 6.833, *P* = 0.0024) at 24 h, 36 h, 48 h, and 60 h, respectively (Fig. 2a–d).

**Fig. 3:**
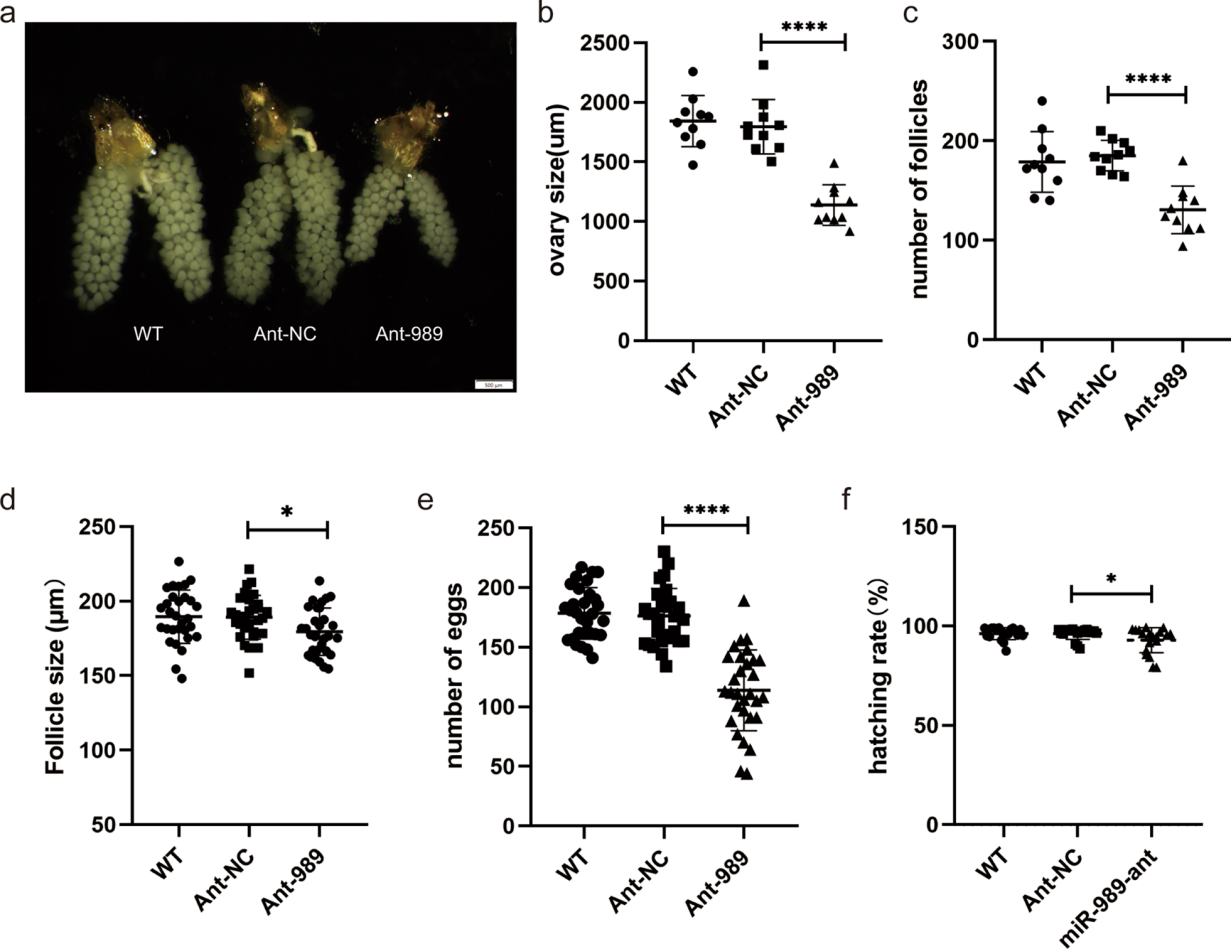
Impact of miR-989 suppression on ovarian development and egg production in female *Culex pipiens pallens*. **a** Ovaries dissected from female mosquitoes microinjected with untreated WT, Ant-NC, or Ant-989. **b** Overall ovarian size per female mosquito. **c** Number of follicles per ovary. **d** Follicle size of ovaries isolated from female mosquitoes. **e** Number of eggs per female mosquito. **f** Hatching rate of eggs. Data represent 30 biological replicates with three technical replicates and are shown as mean ± SD; *P < 0.05; **P < 0.01; ****P < 0.0001

Next, phenotypic manifestations of miR-989-depleted female mosquitoes were observed. Altered ovarian morphology was found in the Ant-989 group (Fig. 3a). We measured the size of entire ovary and single follicle. The number of follicles in each pair of ovaries in female mosquitoes was counted at the same time. Compared with the Ant-NC group, the Ant-989 group displayed smaller ovaries (reduced by 29.48%, *t*_(18)_ = 7.31, *P* < 0.0001) and fewer follicles (reduced by 36.62%, *t*_(18)_ = 6.043, *P* < 0.0001) (Fig. 3b, c). However, changes in the size of each follicle were not significant (reduced by only 5.13%, *t*_(58)_ = 2.45, *P* = 0.0173) (Fig. 3d). Furthermore, Ant-989-treated female mosquitoes showed lower fecundity: miR-989-depletion caused a significant reduction (reduced by 35.37%, *t*_(58)_ = 8.319, *P* < 0.0001) in the number of eggs compared with that in the Ant-NC groups (Fig. 3e). In Ant-989-treated females, blood-sucking behavior, blood digestion processes, and egg incubation (Fig. 3f) were not affected, and these females exhibited no other undesirable traits compared with the control.

### RNA-seq analysis of the ovarian response to miR-989 suppression in *Culex pipiens pallens*

**Fig. 4:**
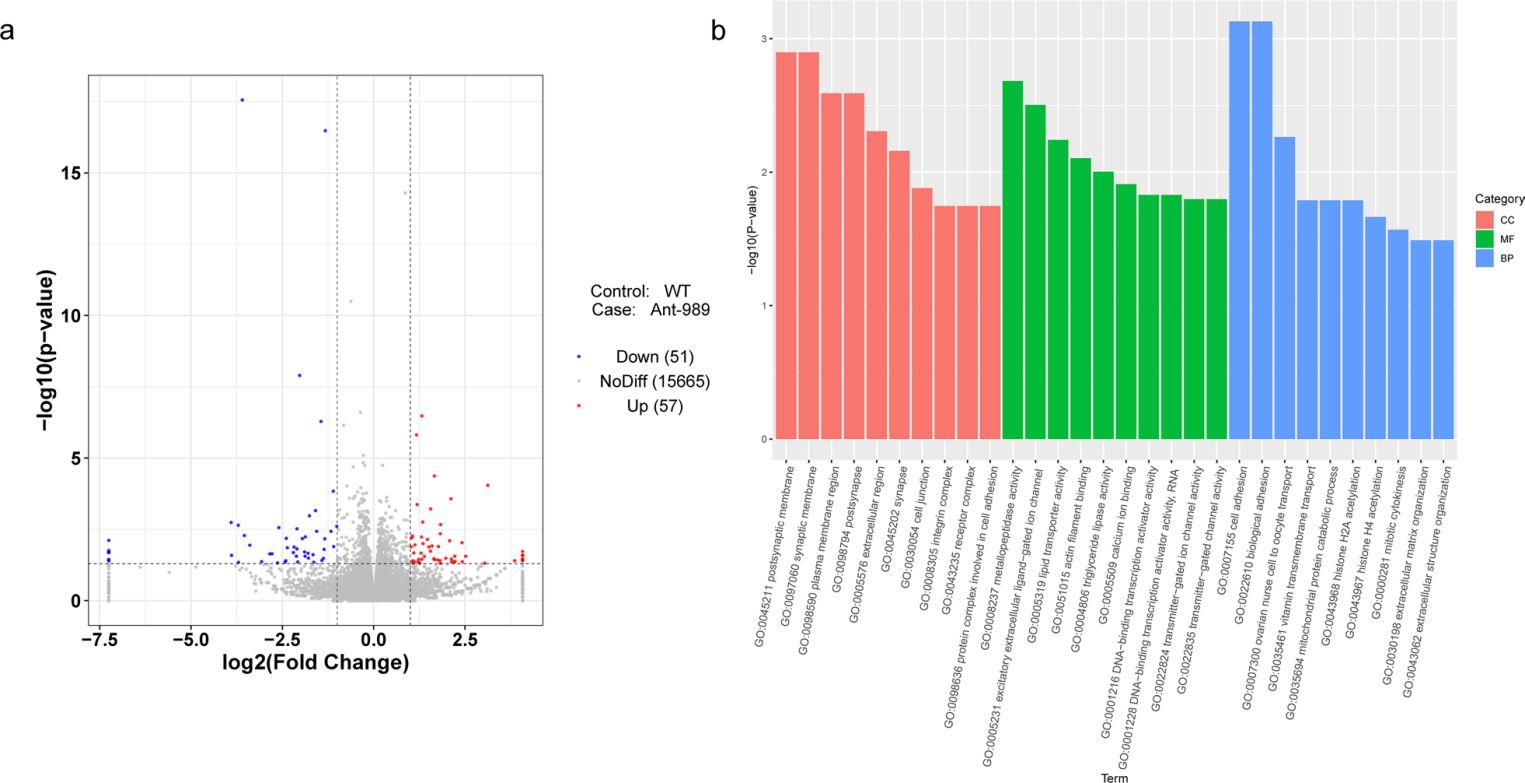
RNA sequencing (RNA-seq) analysis of ovarian response to miR-989 deletion and target gene screening of miR-989. **a** Volcano plot of differentially expressed genes (DEGs) between wild-type (WT) and Ant-989 ovary tissues. Red dots represent upregulated genes, blue dots represent downregulated genes, and gray dots represent non-significant differentially expressed genes. DEGs were identified using a cutoff of *P* < 0.05 and |log2FoldChange (FC)|> 1. **b** Gene ontology of DEGs including three components: biological processes, cellular components, and molecular functions

To determine the molecular mechanism by which miR-989 controls ovarian development, we conducted RNA-seq research on the ovarian tissues of female mosquitoes after the miR-989 knockdown. We collected ovaries from the wild-type and Ant-989-injected mosquitoes at 24 h PBM to construct Illumina sequencing libraries, and three biological replicates were set up for this experiment. miR-989 expression in the ovary was successfully knocked down by 62.06% (Student’s t-tests: *t*_(4)_ = 4.131, *P* = 0.0145) (Additional file 3: Fig. S1). The total number of reads per sample varied between 41,180,666 and 47,816,156 across the six sequenced samples. More than 70% of the reads mapped to the reference genome of *Cx. quinquefasciatus*, and over 95% of the reads were uniquely matched. (Table  1). We performed principal component analysis (PCA) to investigate the clustering of data based on the WT and Ant-989 groups. All samples were split into two separate groups (Additional file 3: Fig. S2). Differential gene expression analysis revealed a total of 108 DEGs, with 57 upregulated DEGs and 51 downregulated DEGs in the Ant-989-injected mosquitoes (Fig. 4a and Additional file 4: Dataset S1). We show separately 10 annotated genes that were over- or under-expressed (Tables  2, 3).

**Table 3:** Genes downregulated after interfering with miR-989

Gene	Transcript ID	Foldchange (WT/Ant-989)
Cuticle protein	CPIJ001839	14.94535221
Netrin	CPIJ016785	11.64073378
Ficolin-1 precursor	CPIJ006396	10.49301766
Advillin	CPIJ004696	6.197548623
Aminopeptidase	CPIJ008410	5.374404203
Caspase-2 precursor	CPIJ008254	5.152181882
Dimethylaniline monooxygenase	CPIJ013725	4.530973563
Coatomer	CPIJ017272	4.333025008
Glutamate receptor	CPIJ018689	4.288268103
Pancreatic triacylglycerol lipase precursor	CPIJ000186	3.657698821

In the WT and Ant-989 groups, based on gene ontology (GO) pathway enrichment analysis, the ion and signal transduction channels, such as cell junction, synapse, and calcium ion binding, were significantly different, confirming that miR-989 plays an essential role in material and energy transport in the ovary. Furthermore, genes in metabolic pathways such as triacylglycerol lipase activity, metalloproteinase activity, and lipid transporter activity were also altered, suggesting that miR-989 might affect the metabolism of female mosquito ovaries. Deletion of miR-989 also affected the expression of genes related to gene expression regulation, such as DNA-binding transcription activator activity and histone acetylation (Fig. 4b).

### *5-HTR1* is a direct target of miR-989

**Fig. 5:**
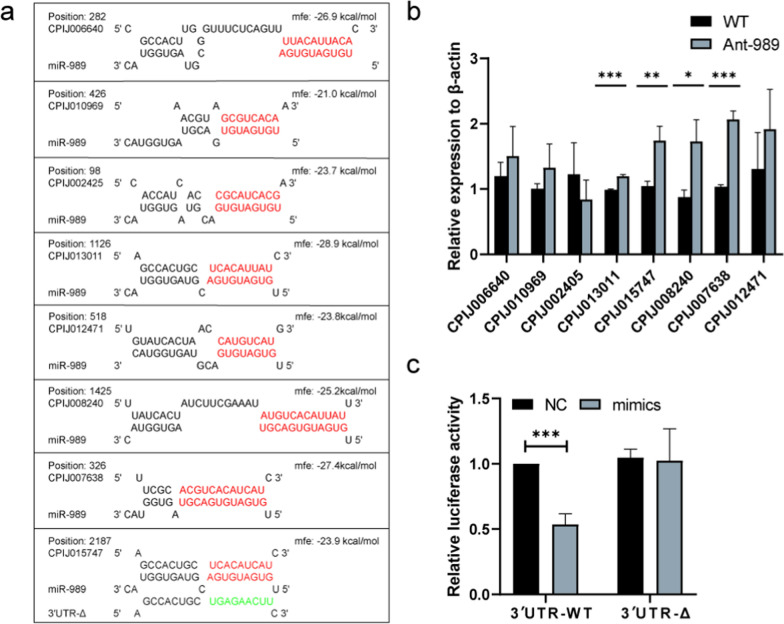
Target genes of miR-989 were predicted by RNA hybrid and screened using quantitative real-time reverse transcription PCR (RT-qPCR, then confirmed using a dual-luciferase reporter assay). **a** Potential miR-989 target sites. Seed sequence of miR-989 and its putative binding sites in the 3′ untranslated regions (UTRs) are marked by red letters; the mutant sequence in the seed region is marked by green letters. **b** Expression levels of candidate target genes in wild-type (WT) and Ant-989 female mosquito ovaries. **c** Dual-luciferase reporter assay for *5-HTR1* in HEK 293 T. The luciferase intensity was reduced by 46.46%. Data represent three biological replicates with three technical replicates and are shown as mean ± SD; *P < 0.05; **P < 0.01; ***P < 0.001. 5-HTR1, secreted *5-HT receptor 1*

The 3'-UTR sequence of the target mRNA is complementary to the mature miRNA seed region sequences (5*'*-end 2–8 nucleotides). We employed the RNAhybrid prediction program to assess the upregulated DEGs in the ovary after a miR-989 knockdown to acquire putative gene targets for miR-989, and eight candidate targets were predicted, including *transcription factor glial cells missing* (CPIJ006640), *unconventional myosin-XVIIIa* (CPIJ002405), *5-HTR1* (CPIJ015747), *carboxylesterase 3 precursor* (CPIJ007638), and *Protease m1 zinc metalloprotease* (CPIJ012471) (Fig. 5a). The transcription levels of these targets were detected after miR-989 intervention in mosquito ovaries. The results displayed that the expression of the *5-HTR1* (increased by 1.67 fold, *t*_(4)_ = 5.2, *P* = 0.0065), *carboxylesterase 3 precursor* (increased by twofold, *t*_(4)_ = 13.32, *P* = 0.0002), and *leucine-rich immune protein* (increased by 1.97 fold, *t*_(4)_ = 4.218, *P* = 0.0135) were upregulated in the Ant-989 group (Fig. 5b). We selected *5-HTR1* as the miR-989 target gene for subsequent experiments because it is the only one that has been shown to potentially regulate reproductive activity in insects [29].

We evaluated the *5-HTR1* 3ʹ-UTR response to miR-989 in vitro to confirm it as an miR-989 target. The *5-HTR1* 3ʹ-UTR containing a predicted miR-989 binding site was cloned downstream of the Renilla luciferase reporter gene. In addition, a construct with a mutant miR-989 binding site inside the 5-HTR1 3ʹ UTR was also produced, resulting in the 5-HTR1 3′UTR-Δ construct. We then transfected them into HEK 293 T cells along with the PGL4.74 plasmid and miR-989 mimic or NC. The luciferase reporter containing the *5-HTR1* 3ʹ UTR-WT sequence decreased the luciferase activity by 46.46% in contrast to the negative control samples (Student’s t-test: *t*_(4)_ = 9.864, *P* = 0.0006). However, regardless of whether they were given NC or the miR-989 mimic, the *5-HTR1* 3′UTR-Δ group's activity remained unchanged (Fig. 5c). Therefore, *5-HTR1* was proved to be a target gene of miR-989 in vitro.

### 5-HTR1 functions in mosquito reproduction

**Fig. 6:**
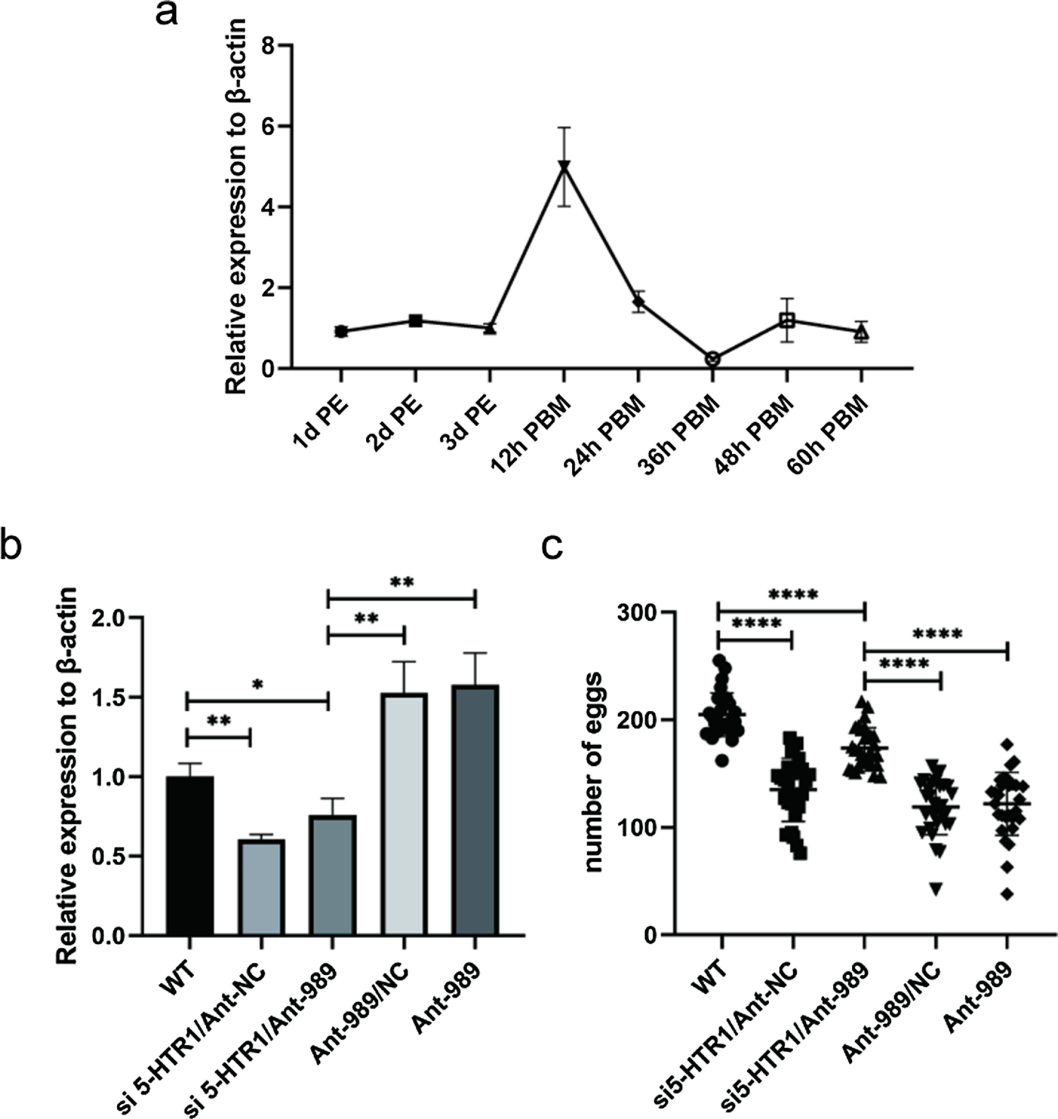
*5-HTR1* RNAi rescues the miR-989 depletion phenotype. **a** Relative expression of the *5-HTR1* mRNA at different developmental periods in mosquito ovaries. **b** Relative expression of *5-HTR1* was quantified from experimental treatments in wild-type (WT); si*5-HTR1*/ antagomir negative control (Ant-NC), coinjection of si*5-HTR1* and Antagomir-NC; si*5-HTR1*/ Ant-989, coinjection of si*5-HTR1* and AntagomiR-989; Ant-989/NC, coinjection of AntagomiR-989 and Negative Control; and AntagomiR-989 injected female mosquitoes. **c** Number of eggs laid per female mosquito in different experimental treatments. Data represent three biological replicates with three technical replicates and are shown as the mean ± SD; *P < 0.05; **P < 0.01; ****P < 0.0001. 5-HTR1, secreted *5-HT receptor 1*

To explore the function of *5-HTR1* in female mosquito reproduction, we first detected the expression levels of *5-HTR1* transcripts in the ovaries of the first reproductive cycle. We extracted total RNA samples from female mosquito ovaries at 1 day, 2 days, 3 days PE and at 12, 24, 36, 48, and 60 h PBM. Expression of *5-HTR1* increased rapidly, by 4.98-fold, at 12 h PBM (Student’s t-test: *t*_(4)_ = 7.072, *P* = 0.0021) and then decreased sharply at 24 h PBM, suggesting a potential role in female mosquito reproduction (Fig. 6a).

To further assess *5-HTR1* as a genuine target gene of miR-989, we carried out phenotypic rescue experiments employing *5-HTR1* RNAi depletion in Ant-989-treated mosquitoes. The negative symptoms brought on by miR-989 silencing were anticipated to be reduced by RNAi of *5-HTR1*. In *Drosophila* and *Ae. aegypti*, this method has been used to successfully restore miRNA mutant phenotypes [16, 30, 31]. In this study, we coinjected 50 pmol of Ant-989 or Ant-NC and 100 ng of si*5-HTR1* or Negative Control (NC) to female mosquito at 12 h PE. The *5-HTR1* transcript level in these Ant-989/si*5-HTR1* mosquito ovaries was significantly lower than that in the Ant-989-injected mosquito ovaries at 24 h PBM (reduced by 51.79%, *t*_(4)_ = 6.213, *P* = 0.0034) (Fig. 6b), In addition, the Ant-989/si*5-HTR1* mosquitoes laid significantly more eggs than Ant-989-injected mosquitoes (increased by 1.43 fold, *t*_(58)_ = 8.205, *P* < 0.0001), reversing the negative effect of miR-989 silencing on egg production (Fig. 6c). Thus, our finding showed that *5-HTR1* is the actual miR-989 target responsible for the undesirable phenotypes observed in female mosquitoes treated with Ant-989.

## Discussion

miRNAs have been shown to play a role in female mosquito reproduction, with many miRNAs being differentially expressed upon vitellogenesis [32, 33]. However, only a limited number of these differentially expressed miRNAs have confirmed target genes or known specific functions. In this study, a highly expressed miRNA, miR-989, was found to have elevated expression after a blood meal in female *Cx. pipiens pallens* ovaries, which was consistent with previous small-RNA sequencing results [21, 22]. However, the role of miR-989 in female mosquito reproduction remains unknown. In our investigation, silencing of miR-989 resulted in smaller ovaries and a reduced number of eggs. In addition, transcriptional screening of miR-989 targets identified *5-HTR1* as a target in vivo, and functional evidence proved that proper mosquito ovarian development requires miR-989-targeted degradation of endogenous *5-HTR1*. Taken together, miR-989 might regulate ovarian development and egg production in female mosquitoes by suppressing *5-HTR1* expression.

miRNAs usually exert different functions through their target genes [34–36]. miR-2/7/13 targets *CYP9J35* and *CYP325BG3* to regulate insecticide resistance in *Cx. pipiens pallens* and targets *Notch* gene to influence the reproduction process of the migratory locust *Locusta migratoria* [13, 37]. miR-989 has been linked to key physiological processes, because miR-989 mutants in *D. melanogaster* have noticeable defects in border cell migration [20], and *doublesex (dsx)* was predicted to be the target gene of miR-989 to regulate sex differentiation in *Bactrocera dorsalis* (Hendel) [38]. miR-989 was shown to target *VgB* using an in vitro dual-luciferase assay in *Ae. aegypti*; however, this was not validated in vivo [23]. Our experiments identified *5-HTR1* as a target gene for miR-989, and the targeted interaction was not only validated by dual-luciferase reporter assay, but also by the fact that the relative expression of *5-HTR1* was upregulated in the miR-989-deletion mosquitoes compared with that detected in the WT. miR-989 reduction resulted in abnormal ovarian development and low fecundity. Taken together, miR-989 might target *5-HTR1* expression to degrade 5-HT and maintain normal ovarian development and egg production in female mosquitoes.

Mosquito ovaries are located dorsolateral in the back of the abdomen and contain primary and secondary follicles, which are composed of three different types of cells: follicular cells for nutrient transport and chorionic villus production, nurse cells for ribosome and mRNA synthesis for the oocyte, and individual oocytes for accumulation of nutrients for the embryo [39]. Oocytes continuously take up yolk protein through receptor-mediated endocytosis after blood aspiration, a process that reaches its peak at 24 h PBM, when follicular cells begin to synthesize eggshell components and secrete them into the perivitelline space between follicular cells and oocytes to form the chorionic villi and eventually the eggshell [4]. Our study found that miR-989 expression was significantly higher in the ovary than in other tissues and was highest at 24 h PBM. In contrast, the expression of miR-989 was almost undetectable at 48 h and 60 h PBM, indicating that miR-989 acts before the formation of the chorionic villus. We found that the main effect of interfering with miR-989 was a decrease in number of follicles and eggs production, with no significant changes in the morphology and size of individual follicles or hatching rate. Therefore, we suggest that miR-989 functions in the early stage of vitellogenesis, mainly to maintain a constant number of follicles, without affecting the subsequent development of follicles.

To understand how miR-989 influences ovarian growth, global gene expression analysis by RNA-seq was carried out following miR-989 depletion in mosquito ovarian. Our findings showed that miR-989 deletion affected numerous genes related to lipid metabolism. *CRALDPCP* (encoded by cellular retinaldehyde-binding protein, CPIJ014229) plays a role in lipid transport and is downregulated in miR-989-deficient mosquito ovaries [40]. *PKG* (encoded by Cgmp-dependent protein kinase, CPIJ006121) has been confirmed to decrease whole body lipid content of *Drosophila* and the *PTS2* (encoding peroxisomal targeting signal 2 receptor, CPIJ014776) mutant in *D. melanogaster* has altered lipid processing [41, 42]. These genes were all activated after miR-989 depletion. Lipid stores are essential for the maturation of the female mosquito oocytes and provide a large amount of energy during the embryogenesis process [43]. Indeed, 90% of the energy required for development in *Culex quinquefasciatus* comes from lipids [44]. Therefore, abnormal expression of genes related to lipid metabolism might lead to impaired ovarian growth. In addition, GO enrichment revealed downregulation of genes associated with lipid transporter activity and triglyceride lipase activity after miR-989 deletion, implying a role of miR-989 on ovarian lipid transport [45]. We also found altered expression of genes related to cell attachment and cell adhesion. Follicular cells adhere to each other through adhesive structures composed of protein multimers, forming a dense cell layer covering the oocyte, while vitellogenin is required to cross the inter-oocyte channels to reach the oocyte by the hemolymph [46–48]. Altered intercellular adhesion might prevent vitellogenin from reaching the oocyte and being absorbed, resulting in impaired ovarian development. Thus, these findings confirm the pivotal function of miR-989 in ovarian development in female mosquitoes.

We demonstrated that miR-989 directly targets *5-HTR1* in vitro and in vivo. miR-989-simulation-binding sites were found in *5-HTR1* using the RNAhybrid program. Deletion of miR-989 via antagomir injection upregulated the mRNA levels of *5-HTR1*, suggesting that miR-989 targets *5-HTR1*. Subsequent dual-luciferase experiments further verified that *5-HTR1* is a real target gene of miR-989 in vitro. Importantly, *5-HTR1* RNAi significantly rescued the phenotype of reduced egg production caused by miR-989 deletion. These findings confirmed that *5-HTR1* is a direct target of miR-989 regulation. 5-HT is an important biogenic amine in insects, which is synthesized in both neural and non-neural tissues, and can be transported and reabsorbed into presynaptic structures via 5-HT receptors [49]. 5-HT exerts different neuromodulatory effects in insects by binding to specific G protein-coupled receptors and regulating major behavioral activities, such as feeding, biological clock, aggregation, learning, and memory [50, 51]. Previously, 5-HT receptors were shown to be associated with female mosquito lipid metabolism and reproduction. In Drosophila, serotonin acts as a neurotransmitter to regulate ILPs [52]. Disruption of the *Ae. aegypti* 5-HT receptor gene using a CRISPR-Cas9 gene editing approach severely affected growth and reproductive processes [29]. Coincidentally, our transcriptome data point to alterations in the expression levels of genes involved in lipid transport in miR-989-deficient mosquitoes. Although the expression levels of these lipid metabolism-related genes did not change significantly after *5-HTR1* RNAi interference, si*5-HTR1* could rescue the Ant-989-induced changes in the expression of these genes. We speculated that *5-HTR1* might be involved in reproduction by affecting lipid transport. Although the function of 5-HT in *Cx. pipiens pallens* remains unknown, RNAi of *5-HTR1* reduced egg production in female mosquitoes. This suggested a potential role in mosquito reproduction; however, further studies are needed to determine how *5-HTR1* affects mosquito reproduction.

Our results suggest that the conserved miR-989 in female mosquito ovaries can regulate the expression of *5-HTR1*, thereby affecting normal ovarian development and egg production. The present study provides a comprehensive resource to determine the intricate procedures involved in miRNA regulation of mosquito reproduction, which will contribute to future research on miRNAs and insect reproduction and will facilitate the creation of effective environmentally friendly pest control methods.

### Supplementary Information

**Additional file 1:Table S1.** Primers used for PCR and vector constructions. **Table S2.** Sequences of the miR-989 antagomir, antagomir-negative control (NC).

**Additional file 2. **Parameters used for HISAT2 software.

**Additional file 3: Figure S1.** Expression levels of miR 989 in WT and Ant-989 female mosquito ovaries. Data presented as mean ± SEM; *P < 0.05. **Figure S2.** Principal component analysis of the ovary RNA-Seq data. A total of six samples were analyzed by RNA-Seq.

**Additional file 4.** DEGs between WT and Ant-989-injected mosquitoes.

## Data Availability

All data generated or analysed in this study are included in this published article.
