# Supplementary material for: MicroRNA-989 targets 5-hydroxytryptamine receptor1 to regulate ovarian development and eggs production in Culex pipiens pallens
Source: Parasit Vectors. 2023 Sep 13;16:326. doi: 10.1186/s13071-023-05957-0 (PMC10498645; doi:10.1186/s13071-023-05957-0)
Supplement: Supplementary file 1 — Additional file 1:Table S1. Primers used for PCR and vector constructions. Table S2. Sequences of the miR-989 antagomir, antagomir-negative control (NC). [file 13071_2023_5957_MOESM1_ESM.pdf]

Table S1. Primers used for PCR and vector constructions

Primers	Forward primer (5' to 3')	Reverse primer (5' to 3')
miR-989	ACACTCCAGCTGGGTGTGATGTGACGTAG	TGGTGTCGTGGAGTCG
U6	GCTTCGGCTGGACATATACTAAAAT	GAACGCTTCACGATTTTGCG
miR-989 SL	CTCAACTGGTGTCGTGGAGTCGGCAATTCAGTTGAGGTACCACT	
5-HT receptor 1	AGTACACAAGCGACTTCTTG	AAGTGGTTATCCCAGGCTAA
β-actin	AGCGTGAACTGACGGCTCTG	ACTCGTCGTACTCCTGCTTGG
5-HTR1 3′UTR-WT	CGAGCTCTGTTACAAGAAAGTACACAA	CCAAGCTTTAATGATGTAAATAGTTATGGTA
5-HTR1 3′UTR-Δ	CGAGCTCTGTTACAAGAAAGTACACAA	CCAAGCTTTAACGTTCTCAATAGTTATGGTA

Table S2. Sequences of the miR-989 Antagomir, Antagomir negative control (NC), miR-989 mimic, negative control, and si5-HTR1 (5-HT receptor 1)

Name	Sense (5' to 3')	Antisense (5' to 3')
miR-989 Antagomir	GUACCACUACGUCACAUCACA	
Antagomir NC (Ant-NC)	CAGUACUUUUGUGUAGUACAA	
miR-989 mimic	UGUGAUGUGACGUAGUGGUAC	ACCACUACGUCACAUCACAUU
Negative control (NC)	UUCUCCGAACGUGUCACGUTT	ACGUGACACGUUCGGAGAATT
Si *5-HT receptor 1*	GACCAUGCAACUACCUACUTT	AGUAGGUAGUUGCAUGGUCTT
