# Supplementary material for: MicroRNA-989 targets 5-hydroxytryptamine receptor1 to regulate ovarian development and eggs production in Culex pipiens pallens
Source: Parasit Vectors. 2023 Sep 13;16:326. doi: 10.1186/s13071-023-05957-0 (PMC10498645; doi:10.1186/s13071-023-05957-0)
Supplement: Supplementary file 2 — Additional file 2. Parameters used for HISAT2 software. [file 13071_2023_5957_MOESM2_ESM.docx]

--mp <int>,<int> max and min penalties for mismatch; lower qual = lower penalty <6,2>

--sp <int>,<int> max and min penalties for soft-clipping; lower qual = lower penalty <2,1>

--no-softclip no soft-clipping

--np <int> penalty for non-A/C/G/Ts in read/ref (1)

--rdg <int>,<int> read gap open, extend penalties (5,3)

--rfg <int>,<int> reference gap open, extend penalties (5,3)

--score-min <func> min acceptable alignment score w/r/t read length (L,0.0,-0.2)
