# Supplementary material for: MicroRNA-989 targets 5-hydroxytryptamine receptor1 to regulate ovarian development and eggs production in Culex pipiens pallens
Source: Parasit Vectors. 2023 Sep 13;16:326. doi: 10.1186/s13071-023-05957-0 (PMC10498645; doi:10.1186/s13071-023-05957-0)
Supplement: Supplementary file 3 — Additional file 3: Figure S1. Expression levels of miR 989 in WT and Ant-989 female mosquito ovaries. Data presented as mean ± SEM; *P < 0.05. Figure S2. Principal component analysis of the ovary RNA-Seq data. A total of six samples were analyzed by RNA-Seq. [file 13071_2023_5957_MOESM3_ESM.pdf]

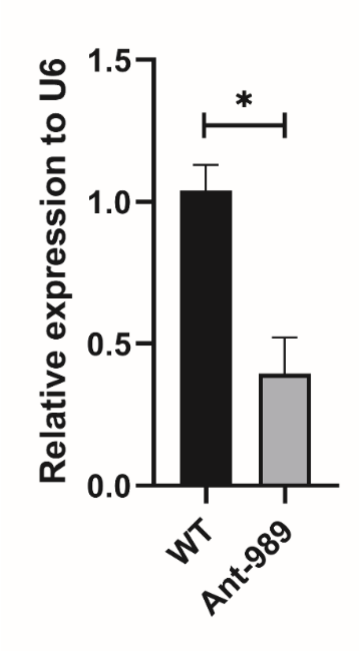

Fig. S1 Expression levels of miR 989 in WT and Ant-989 female mosquito ovaries. Data presented as mean ± SEM; *$P < 0.05$.

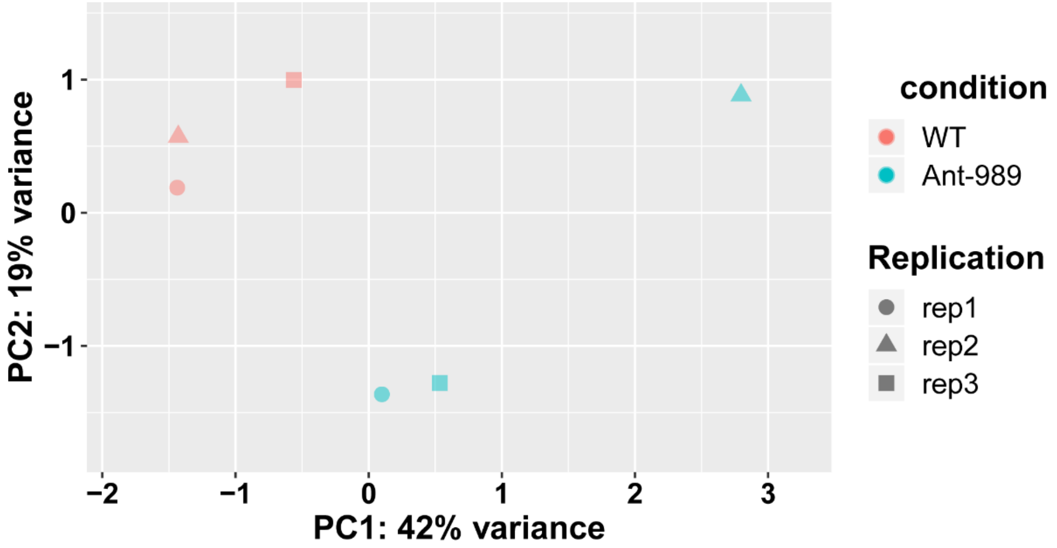

Fig. S2 Principal component analysis of the ovary RNA-Seq data, A total of 6 samples were analyzed by RNA-Seq.
